# Function-based risk reduction intervention for lifestyle-related disorders among inactive 40-year-old people: a pilot randomised controlled trial

**DOI:** 10.1186/s12889-024-20301-6

**Published:** 2024-10-13

**Authors:** Lena Bornhöft, Daniel Arvidsson, Anna Bergenheim, Mats Börjesson, Jonatan Fridolfsson, Margareta Hellgren, Lena Nordeman, Maria E. H. Larsson

**Affiliations:** 1 Unit of Physiotherapy, Department of Health and Rehabilitation, Institute of Neuroscience and Physiology, Sahlgrenska Academy, University of Gothenburg, Gothenburg, Sweden; 2 Research, Education, Development, and Innovation Primary Health Care, Region Västra Götaland, Gothenburg, Borås, Vänersborg and Skövde, Sweden; 3 Närhälsan Torslanda Rehabilitation Clinic, Primary Care Rehabilitation, Region Västra Götaland, Torslanda, Sweden; 4 Center for Health and Performance, Department of Food and Nutrition and Sports Science, University of Gothenburg, Gothenburg, Sweden; 5 Department of Molecular and Clinical Medicine, Sahlgrenska Academy at University of Gothenburg, Gothenburg, Sweden; 6 Center for Lifestyle Intervention, Department of MGAÖ, Sahlgrenska University Hospital, Region Västra Götaland, Gothenburg, Sweden; 7 General Practice - Family Medicine, School of Public Health and Community Medicine, Institute of Medicine, Sahlgrenska Academy, University of Gothenburg, Gothenburg, Sweden; 8 The Skaraborg Institute, Skövde, Sweden

**Keywords:** Lifestyle-related disorders, Prevention, Physical activity, Function, Risk profile

## Abstract

**Background:**

Interventions for preventing or reducing the development of lifestyle-related disorders should be investigated as these conditions are becoming increasingly prevalent and having large effects on quality of life and life expectancy globally. The aim of this pilot study was to prepare for a full-scale randomised controlled trial by evaluating the short-term changes resulting from a function-based preventive intervention aimed at lifestyle-related disorders on a small group of physically inactive 40-year-old people. Change in objectively measured physical activity, functional capacity according to a risk profile, and goal attainment were main outcomes.

**Methods:**

Participants (*n* = 27) underwent functional examinations including tests of fitness, strength, mobility, balance, and posture as well as standard medical examinations including weight measures, blood pressure and blood tests and were randomised to two groups. The intervention group (*n* = 15) received feedback from all the examinations and lifestyle counselling based on a functional profile. The control group (*n* = 12) received feedback only from the standard medical examination. Follow-up was at 3–4 months. Changes in physical activity measured with accelerometers, functional levels on the functional profile, goal attainment and subjective assessments of health-related quality of life, motivation, function, and physical activity were examined, as were standard medical parameters.

**Results:**

Change in mean time in moderate or more intense physical activity was 9 min higher in the intervention group (95% confidence interval -6.35, 24.51) and change in sedentary time was 42 min lower (-95.24, 11.32). The intervention group showed a higher increase in motivation for change 1.58 on 10-point scale (0.20, 2.97) and indicated more improvement on the functional risk levels concerning fitness (-0.06, 0.90). Correlation between objectively measured and self-assessed physical activity and function increased after the intervention. Most participants in the intervention group achieved some or all of their goals.

**Conclusions:**

This small-scale pilot intervention with functional examinations and lifestyle counselling showed positive tendencies for change in short-term physical activity level. It seemed to lead to better understanding of personal functional capacity and increased motivation for lifestyle changes. Setting and fulfilling meaningful goals for lifestyle-related changes seemed to influence levels on the functional profile in positive directions. Research on larger and more diverse populations will be necessary to better understand the implications of the intervention.

**Trial registration:**

ClinicalTrials.gov: NCT05535296 first posted on 10/09/2022.

**Supplementary Information:**

The online version contains supplementary material available at 10.1186/s12889-024-20301-6.

One definition of lifestyle-related disorders (LRD) is those diseases and disorders “whose occurrence is primarily based on the daily habits of people and are a result of an inappropriate relationship of people with their environment” [1]. While metabolic and cardiovascular diseases are clearly included in this description, another large group of disorders are sometimes overlooked. Disorders affecting the musculoskeletal system are among the most prevalent conditions affecting health and lifestyle of both the Nordic and the global populations [2, 3]. Many musculoskeletal disorders are lifestyle-related and are interconnected with metabolic and cardiovascular diseases [4]. For the individual, it can vary which type of health problem arises first. However, in contrast to metabolic and cardiovascular disease, musculoskeletal problems are often painful in all stages of the disorder, which can easily lead to avoidance of particular movements, to general physical inactivity and eventually contribute to overweight, hypertension and other related metabolic and cardiovascular conditions [4]. Prevention of musculoskeletal problems may, therefore, be instrumental in prevention of other LRDs.

Length of life has been steadily increasing and, as prevalence of LRD increases with age, more years are spent in poor health [2]. LRD can theoretically be prevented or reduced but it is unclear how this result can best be achieved. Many primary prevention programs have generally good effects [5, 6], as do secondary prevention programs [7]. In Sweden, such programs often focus on cardiovascular and metabolic diseases [5–7]. Primary prevention focusing on musculoskeletal disorders is a field of interest which has largely been neglected [8]. Many health and behavioural aspects, such as physical activity level, are important and common factors to all LRD. However, there are also aspects which are primarily musculoskeletal, and which are seldom followed by health care.

A variety of functional outcomes are correlated to prevalence of LRD but are seldom measured in pre-symptomatic populations or even in risk-populations. Associations between specific physical functions such as fitness level or balance problems and development of specific LRD or groups of LRD such as cardiovascular disease and falls and fractures in the elderly are well-known [9, 10]. However, knowledge about the relation between other functions, such as strength or mobility, and LRD is not as intuitive or widespread, but both these functions correlate to early mortality and, in some cases, extent and intensity of disease symptoms [11–13]. It is conceivable that screening of physical function and lifestyle counselling specifically directed to physical function can play a part in the prevention of musculoskeletal disorders and, in the long term, even prevention of LRD in general.

Physical inactivity has been increasing rapidly during the latest decades, especially in high-income countries [14]. There is extensive evidence that physical activity and exercise can be used with good effect as part of the treatment plan for many LRDs [15, 16]. Physical activity level measured objectively with accelerometers is a useful complement to self-assessment in both research and clinical practice giving reliable data that can be used to measure change [17].

Pilot-testing new interventions on a small scale can give vital information to the planning of larger studies without the time and resource demands needed to find statistically significant and clinically relevant effects [18].

Our research group developed a standardised protocol for testing physical function based on published literature in the field and examined the feasibility of using the protocol as part of a composite preventive intervention regarding 40-year-old physically inactive people [19]. The protocol included a sub-maximal ergometer fitness test and common clinical tests of strength, balance, mobility and posture. Participants were examined according to the protocol and a functional profile was compiled. Feedback and lifestyle counselling were then based on the functional profile. The participants were positive to being screened for functional capacity and felt they could use the results of the intervention to steer lifestyle choices. The intervention was found feasible in terms of time, resources and examiner and participant experiences [19].

The aims of the current study were to prepare for a full-scale randomised controlled trial (RCT) by evaluating the short-term changes resulting from a function-based preventive intervention aimed at lifestyle-related disorders on a small group of physically inactive 40-year-old people and to enable a power calculation for the full-scale RCT. The study also aimed to investigate the relationship between self-assessed and objectively measured physical function and activity level in the target group to test the presumption that it was low and in need of being addressed which was a major part of the rationale for the development of this intervention.

## Methods

The procedure for the pilot study has been described in detail in an earlier article on development of the intervention and testing its feasibility in a primary care environment [19]. Self-assessed physically inactive 40-year-old people were recruited from the general population in an area with diverse socioeconomic conditions and examined at inclusion and again after 3–4 months. A list of 40-year-olds in the defined area was procured from Statistics Sweden in order of birthdate and invitation letters were sent out consecutively from the list until a sufficient number of people had agreed to participate. Interested parties contacted the project leader after they received the invitation letter. They were screened by telephone to ascertain that inclusion and exclusion criteria were fulfilled. Besides age and physical activity level requirements, participants were expected to have normal general mobility and no ongoing serious illness or condition which would affect their ability to participate in the study. Full details of inclusion and exclusion criteria have already been published [19]. Follow-up at three months was chosen for this pilot study to give the participants enough time to make some lifestyle changes without prolonging the study period unduly as the study was designed to provide better preliminary understanding of the intervention rather than to achieve statistically significant change in outcomes. Both functional and medical examinations were performed. Participants were randomised to intervention or control groups after the inclusion examinations. One-to-one randomisation was prepared in advance using a computer algorithm, sealed opaque envelopes and blocks of four by a person not active in the project. The intervention group received feedback from both parts of the examination and was given support in setting realistic goals and making plans to achieve these goals based on a functional profile calculated after the examinations. A nurse gave feedback on the results of the blood tests, blood pressure, and anthropometric measures in a single visit after each of the inclusion and follow-up examinations. A physiotherapist gave feedback on the results of the functional tests, explained the functional profile, and supported the setting of goals in a single visit after each of the inclusion and follow-up examinations. The examined functional dimensions were fitness, strength in upper extremity, lower extremity and trunk muscles, balance, mobility and posture. At least two functional tests were included in each dimension. Functional levels were based on proportional deviation from population norms or recommended values according to published formulae [19]. The control group received only feedback from the nurse regarding the standard medical examination.

Change in objectively measured physical activity, functional capacity according to a functional profile, and goal attainment were examined with difference in daily minutes in moderate or more intense physical activity being the primary outcome. Other outcomes regarding health-related quality of life, self-assessed physical activity and function, motivation for change, risk for developing chronic pain, mental health and standard medical variables were also examined to achieve a better basic understanding of which effects this new intervention can have and to aid planning of larger studies [19–26].

All participants wore the Axivity AX3 accelerometer (Axivity Ltd, UK) in an elastic belt around the waist on the right side 24 h a day for 7 days both at inclusion and follow-up. Raw triaxial data was processed to physical activity intensity (mg) using the 10 Hz frequency extended method (FEM) and calibrated cut-points were applied to assess time spent in sedentary, light, moderate, vigorous and very vigorous physical activity [27]. Bedtime was recorded by the participants in a diary. Non-wear time was defined as 60 min of zero accelerometer output with allowance of up to 2 min of interruptions. A valid day was defined as at least 10 h and a valid measurement having at least 4 days. Even if the participants were instructed to wear the accelerometer for 24 h a day, it is possible that they might take it off and forget to put it on again. As our primary outcome is mean daily moderate-to-very vigorous physical activity and as it was observed in a previous population study of individuals wearing accelerometer daily only, that 10 h was a good compromise between sufficient wear time and sample size, we chose 10 h as the minimal wear time [28]. Participants and clinical examiners were blinded as to the accelerometer results.

Secondary outcomes were goal attainment and changes in risk levels for each functional dimension. Changes in functional outcomes, health-related quality of life, self-assessed physical activity level and sedentary behaviour, risk for developing chronic pain, motivation for change, mental health, and smoking habits, body mass index, waist circumference, blood pressure, glucose, total cholesterol, triglycerides, low density lipoproteins, high density lipoproteins were also examined. Participants were asked to estimate their functional capacity regarding fitness, strength in upper and lower extremity, balance and walking ability before each examination occasion.

*Statistical analysis:* Descriptive statistics and 95% confidence intervals (95% CI) are used to describe the results as are recommended for pilot studies [29]. Correlations between self-assessed physical activity and functional levels and objectively measured levels were calculated with Spearman’s correlation coefficient.

## Results

**Fig. 1:**
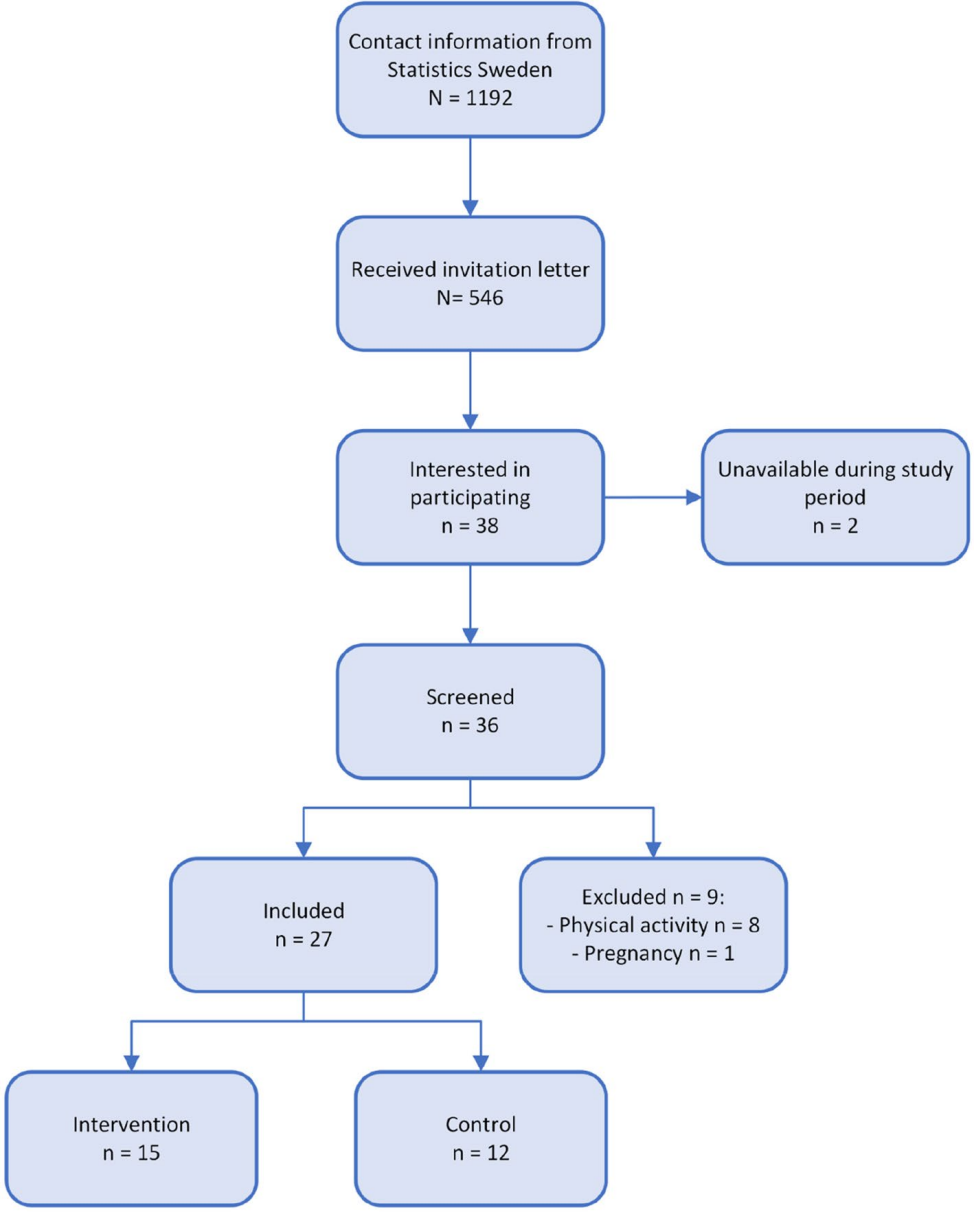
Flowchart

A total of 546 people received an invitation letter, 38 registered interest in participating and 27 were included in the pilot study (Fig. 1). Two people in the intervention group were lost to follow-up because of personal reasons unconnected to the project. Another two, also from the intervention group, were unable to participate in complete examinations at follow-up because of health reasons. Inclusion examinations were made in September–October 2022 and follow-up in January–February 2023.

Participant characteristics are shown in Table 1. Demographics were fairly comparable between groups at baseline although with some differences in distribution of education and income categories. Participants were generally healthy with few known diseases or mental health problems, and few were smokers (Additional files 1 and 2).

**Table 1:** Demographic characteristics of participants at baseline

	**INTERVENTIONN (%)** ***n* = 15**	**CONTROLN (%)** ***n* = 12**	**95% Confidence intervals**
Sex			
N_MALE_	8 (53%)	6 (50%)	-0.38, 0.45
Education			
N_SECONDARY SCHOOL_	1 (7%)	0 (0%)	-0.78, 0.05
N_GYMNASIUM_	6 (40%)	2 (17%)	
N_UNIVERSITY_	8 (53%)	10 (83%)	
Main activity			
N_PHYSICAL WORK_	2 (13%)	1 (8%)	-0.43, 0.43
N_NON-PHYSICAL WORK_	12 (80%)	10 83%)	
N_SCHOOL_	0 (0%)	1 (8%)	
N_OTHER_	1 (7%)	0 (0%)	
Income			
N_LOW_	3 (20%)	1 (8%)	-0.96, 0.19
N_MEDIUM_	6 (40%)	3 (25%)	
N_HIGH_	6 (40%)	8 (67%)	
Residence			
N_URBAN_	12 (80%)	12 (100%)	-0.43, 0.03
N_RURAL_	3 (20%)	0 (0%)	
Country of birth			
N_SWEDEN_	8 (53%)	8 (67%)	-0.54, 0.27
N_NOT SWEDEN_	7 (47%)	4 (33%)	
Raised in			
N_SWEDEN_	10 (67%)	9 (75%)	-0.46, 0.29
N_NOT SWEDEN_	5 (33%)	3 (25%)	
Civil state			
N_SINGLE_	4 (27%)	3 (25%)	-0.38, 0.35
N_COHABITATING_	11 (73%)	9 (75%)	

Table 2 shows the distribution of time spent at the different intensity levels for physical activity defined from accelerometer data. Change between inclusion and follow-up in mean time in moderate or more intense physical activity was 9 min higher in the intervention group (95% CI -6.35, 24.51) and change in sedentary time was 42 min lower (95%CI -95.24, 11.32).

**Table 2:** Change in physical activity between inclusion and 3-month follow-up measured with accelerometers

Activity	Intervention *n* = 13 Mean daily minutes (SD)			Control *n* = 12 Mean daily minutes (SD)			95% confidence intervals (between-groups change)
	*Inclusion*	*Follow-up*	*Mean change*	*Inclusion*	*Follow-up*	*Mean change*	
**BED**	472 (63)	483 (58)	14 (46)	494 (43)	493 (56)	-1 (62)	-30.93, 60.60
**SEDENTARY**	712 (76)	700 (52)	-13 (63)	712 (62)	740 (70)	29 (65)	-95.24, 11.32
**LIGHT PA**	153 (35)	146 (31)	-9 (29)	133 (39)	123 (37)	-10 (23)	-21.04, 22.23
**MODERATE PA**	87.6 (19.0)	84.9 (18.7)	-5.0 (17.0)	78.4 (21.3)	65.0 (20.4)	-13.4 (18.0)	-6.12, 22.94
**VIGOROUS PA**	1.6 (2.6)	1.7 (2.6)	-0.1 (3.5)	0.9 (0.8)	0.5 (0.5)	-0.4 (0.7)	-1.84, 2.48
**VERY VIGOROUS PA**	0.2 (0.4)	0.4 (1.2)	0.2 (1.3)	0.2 (0.4)	0.1 (0.1)	-0.1 (0.4)	-0.48, 1.18
**M + V + VV PA**	89.4 (20.2)	87.1 (19.7)	-4.9 (19.2)	79.5 (22.0)	65.6 (20.7)	-13.9 (18.1)	-6.35, 24.51

*SD* Standard deviation, *PA* Physical activity, *M* + *V* + *VV* Moderate + vigorous + very vigorous

**Fig. 2:**
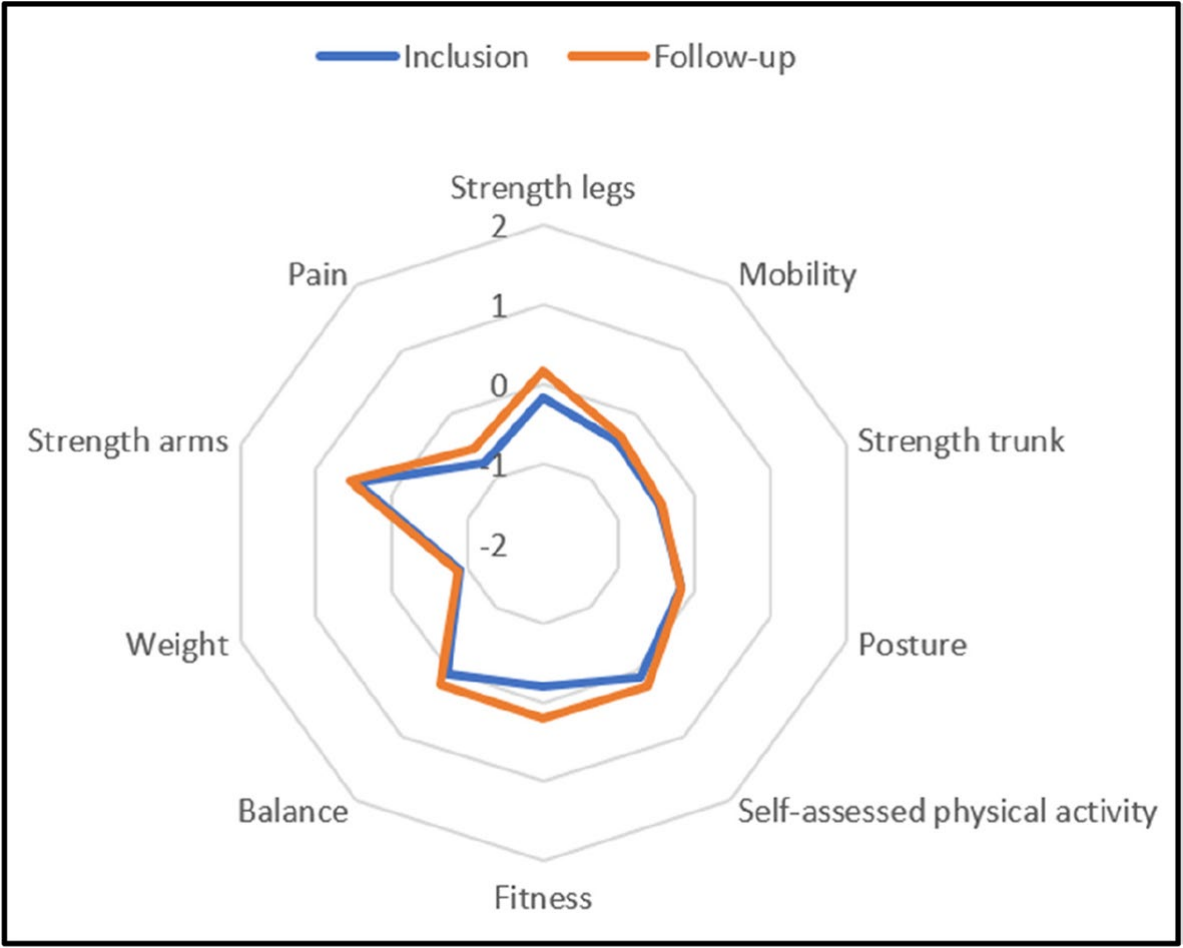
Functional profile for intervention group

**Fig. 3:**
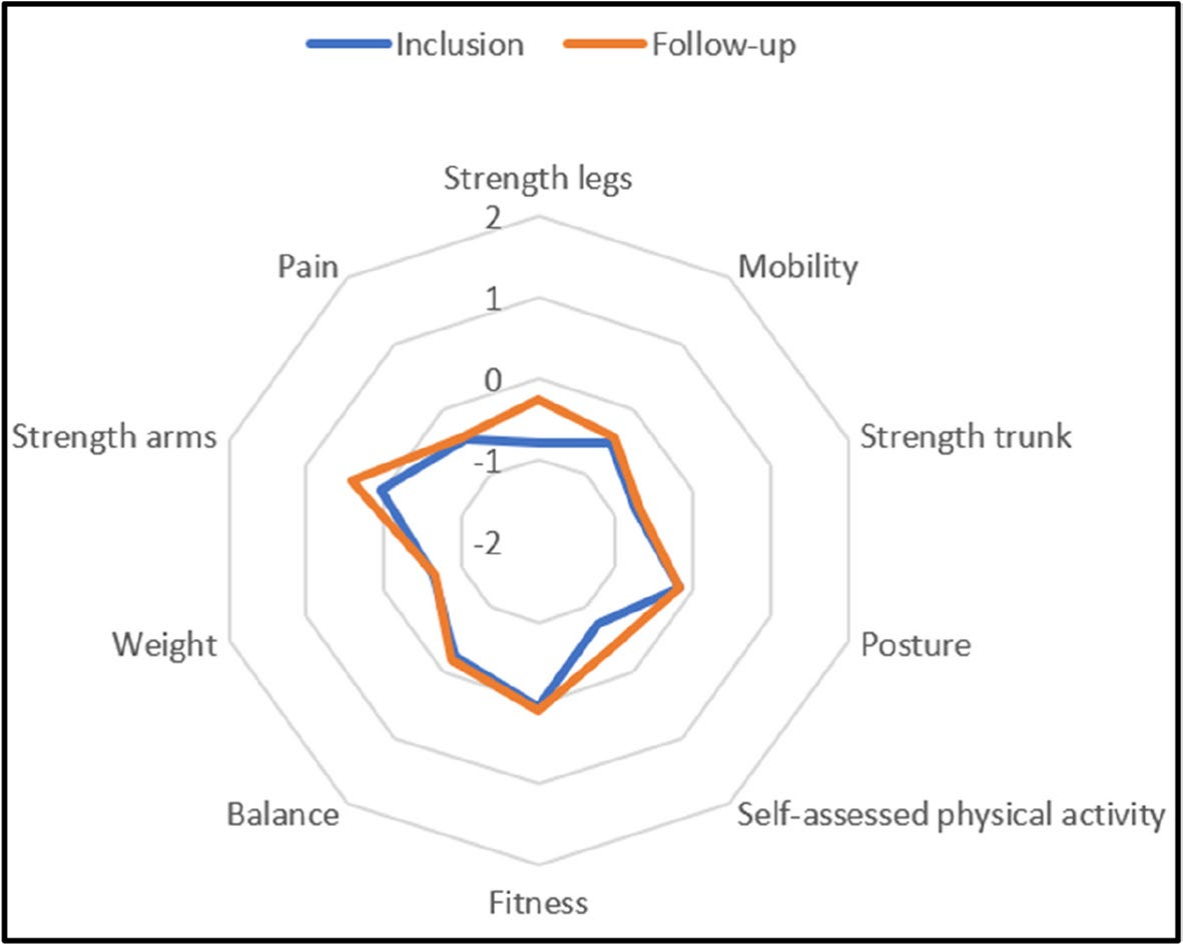
Functional profile for control group

**Fig. 4:**
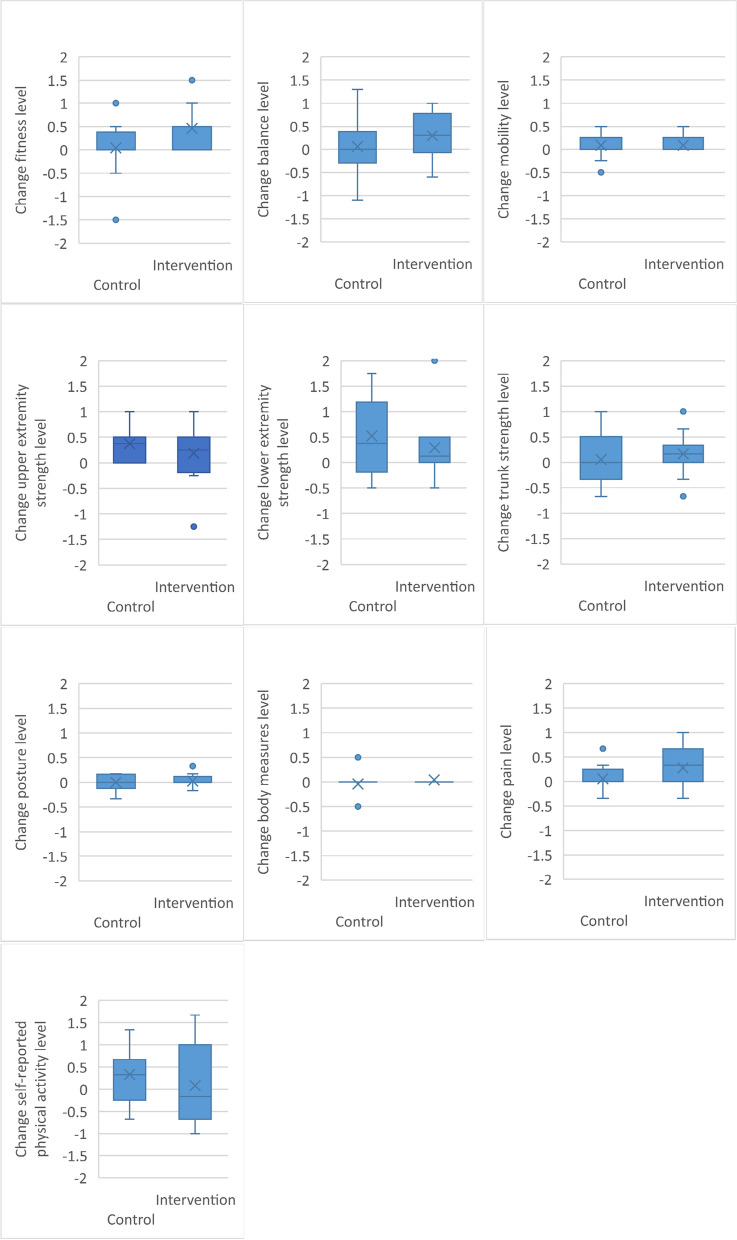
Boxplots showing change in functional dimensions for intervention and control groups

Functional levels were calculated for the following dimensions: fitness, strength in upper extremity, lower extremity and trunk muscles, balance, mobility, posture, weight measures, self-assessed physical activity, and pain according to separately published procedures [19]. A functional level of “0” indicates recommended values, if there are any, otherwise population norms. Men and women receive point values in comparison to sex-specific cut-off levels for each test which are then combined to a point value for each dimension. There were generally small positive changes over time in functional levels for both groups and almost all dimensions, except for weight and posture in the control group (Figs. 2, 3 and 4). The largest difference between groups in change between inclusion and follow-up was in fitness level, favouring the intervention group.

**Fig. 5:**
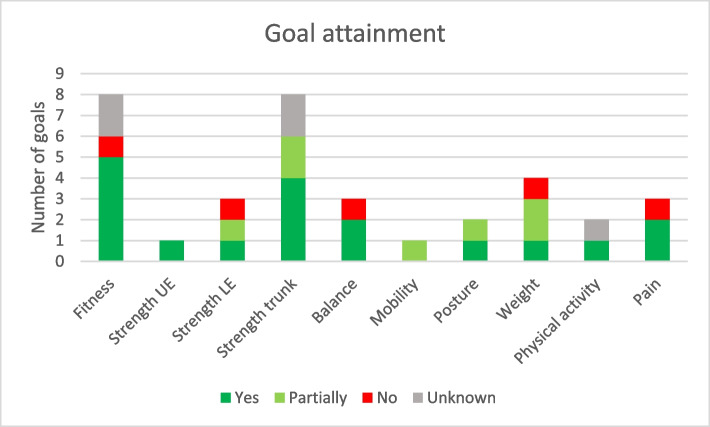
Goal attainment per dimension in the functional profile

**Fig. 6:**
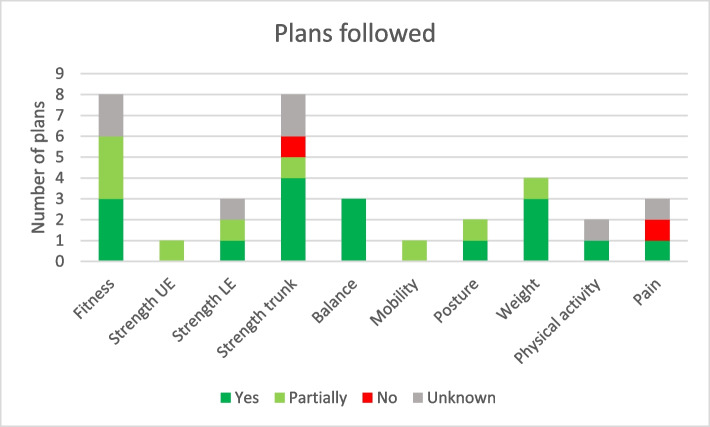
How individual plans for achieving goals were followed, per dimension in the functional profile

Participants in the intervention group were encouraged to set attainable goals and make plans for achieving them after the inclusion examinations. Goals were individual. Participants were encouraged to focus on dimensions where they had earned low scores, but they made their own choices and set as many goals as they felt appropriate. Goal attainment was investigated after the follow-up examinations. The most frequent goals concerned fitness (23% of total goals) and strength in trunk muscles (23%), followed by weight (11%). Of the total 35 set goals, 71% were achieved, at least partially, and, in 74% of the cases, participants followed their original plans either wholly or partially (Figs. 5 and 6).

Results from the questionnaires regarding health-related quality of life, self-assessed physical activity and sedentary behaviour, risk for developing chronic pain, motivation for change and mental health at inclusion and follow-up are shown in Supplementary Table 1, Additional file 1 for both intervention and control groups. Self-reported illnesses were stated at inclusion. No participants reported diabetes, heart disease or chronic obstructive pulmonary disorder. Participants with musculoskeletal problems did not report them as illness but it became evident during the study that many participants had pain or function-related problems. The physical activity questionnaires Saltin-Grimby Physical Activity Level Scale (SGPALS) and the Swedish National Board of Health and Welfare Physical Activity (NBHW-PA) questionnaire showed significantly higher values in the intervention group than in the control group at inclusion. The change in motivation for making lifestyle changes to improve health was significantly higher in the intervention group at follow-up (between-groups difference for mean change was 1.58 (on 10-point scale) (95% CI 0.20, 2.97)).

Results of individual functional tests for both groups at both time points can be found in Supplementary Table 2, Additional file 2. Both groups showed slight improvement on the majority of tests at follow-up. Results on the Plank test improved significantly in the intervention group compared to the control group (between-groups difference for mean change was 18.4 s (95% CI 4.24, 32.58).

The correlations between self-assessed function and physical activity level and objectively measured values were investigated at inclusion and follow-up (Table 3). The correlations between self-assessed and objectively measured fitness level, strength in the lower extremity and physical activity level were particularly low at inclusion. The estimates from both groups show generally better correlation with objective values at follow-up than at inclusion, with exception for physical activity in the intervention group and walking ability in the control group. None of the participants received feedback about their objectively measured physical activity level after inclusion but the intervention group received feedback about physical activity level based on self-reported questionnaires after the inclusion examinations.

**Table 3:** Correlations between self-assessed and objectively measured function and physical activity

**Self-assessed**	**Objective measurements/risk levels**					
	**Intervention**		**Control**		**All**	
	*Inclusion*	*Follow-up*	*Inclusion*	*Follow-up*	*Inclusion*	*Follow-up*
**Fitness**^**a**^	-0.05	0.31	0.14	0.15	-0.01	0.25
**Strength UE**^**b**^	0.41	0.85***	0.51	0.71**	0.46*	0.75***
**Strength LE**^**c**^	-0.26	0.43	0.09	0.45	-0.05	0.47*
**Balance**^**d**^	0.04	0.59	0.29	0.62*	0.18	0.55**
**Walking ability**^**a**^	0.39	0.77**	0.61*	0.34	0.48*	0.53**
**NBHW PA**^**e**^	0.46	-0.10	-0.09	0.19	0.29	0.17
**SGPALS**^**e**^	0.08	-0.29	-0.10	0.43	0.13	0.18
**SED-GIH**^**f**^	0.05	0.36	0.72**	0.79**	0.35	0.67***

^*^p < 0.05

^**^p < 0.01

^***^p < 0.001

^a^In relation to Risk level fitness

^b^In relation to Risk level strength upper extremity

^c^In relation to Risk level strength lower extremity

^d^In relation to Risk level balance

^e^In relation to moderate + vigorous + very vigorous physical activity measured by accelerometers

^f^In relation to sedentary time measured with accelerometers. Spearman’s correlation coefficients are shown, in boldface where statistically significant

The changes in results of the blood tests and measures of weight, body-mass index (BMI), waist circumference and blood pressure between inclusion and follow-up were mostly small and all between-group differences were non-significant (Supplementary Table 3, Additional file 3). In the intervention group, there was less than 1% change for weight, BMI, waist circumference and diastolic blood pressure, while systolic blood pressure increased by 4.1%. In the control group, there was less than 1% change for weight, BMI and systolic blood pressure. Waist circumference increased by 1.2% and diastolic blood pressure decreased by 2.1%. Values on all the blood tests increased for the intervention group (glucose 6.2%, total cholesterol 5.8%, triglycerides 5.4%, LDL/HDL ratio 4.4%). Values for the control group also increased except for triglycerides which decreased (glucose 0.4%, total cholesterol 9.6%, triglycerides -12.8%, LDL/HDL ratio 6.1%). Of the three smokers in the study (two in the intervention group, one in the control group), one in the intervention group quit smoking between inclusion and follow-up.

Adverse events: One participant who had fallen ill between inclusion and follow-up and was not fully recovered at examination time needed to discontinue the functional examination at follow-up due to exertion-related symptoms.

## Discussion

An intervention consisting of functional examinations, lifestyle counselling and goalsetting based on a visually understandable functional profile was investigated in a pilot study. Tendencies were seen for the intervention group to have more physical activity of at least moderate intensity and less sedentary behaviour than the control group at follow-up. Motivation for making lifestyle changes seemed to increase in the intervention group despite high initial levels in both groups. Clear differences were seen between self-assessed and objectively measured function and physical activity level at inclusion. The intervention seemed to lead to more realistic estimates of function. No feedback was given concerning objectively measured physical activity after the inclusion examinations and the correlation with self-reported physical activity did not improve. Both groups were slightly below normal or recommended levels for most dimensions on the functional profile at inclusion and improved somewhat at follow-up. Most participants in the intervention group were able to set reasonable goals for making lifestyle changes and to achieve their goals.

The change in physical activity pattern between groups during the study period was in a consistent direction, with a mean difference between groups of 9 min daily regarding moderate and more intense physical activity and 42 min regarding sedentary behaviour in the intervention group’s favour. However, the pilot study was underpowered to reach statistical significance. The intervention group reduced their moderate and more intense physical activity between the inclusion examinations at the beginning of the fall and the follow-up in the middle of the winter. However, the reduction was less than in the control group. The control group also increased their sedentary time between the two time points which the intervention group did not. As some of the participants pointed out, the time of year can have a large effect on how active people are [19]. The control group was less active in the winter while the intervention group seemed to maintain their “autumn level” of activity to a higher degree.

Both groups spent on average more than 10 h a day sitting still at inclusion. This indicates that we succeeded in recruiting the target group of physically inactive 40-year-old people, despite difficulties that many participants had with accounting for their physical activity level [19].

Fitness level is strongly correlated to risk for cardiovascular disease and the metabolic syndrome and is considered to be one of the most important factors in reducing impacts of these conditions [9, 30, 31]. Helping people to understand how fit they are and how much or little exertion is needed on their own part to achieve a better fitness level may be important in motivating and maintaining lifestyle changes. There was no correlation between self-assessed and objectively measured fitness level at inclusion. However, at follow-up, the correlation between self-estimated fitness and walking ability and objectively measured fitness level was markedly better in the intervention group than in the control group. This can be interpreted as a more realistic understanding of personal functional capacity. Lack of understanding for personal function may limit some people’s efforts to improve health. Increasing fitness was one of the most common goals set by the intervention group. Of the functional levels, change in fitness level showed the largest between-groups differences.

Besides improving fitness, several people set goals of improving strength in trunk muscles. Overall, many participants were weak in some or all the measured trunk muscle groups at inclusion. The intervention group increased their strength in the Plank and Supine Bridge tests but not in the Back Endurance test. The change in Plank test was significantly better in the intervention group compared to the control group at follow-up despite the small group size. Functional testing may inspire training of tested functions. While the first two tests are easy to perform as home exercises, Back Endurance needs to be adjusted for home training. Perhaps people with weak back muscles would gain from physiotherapist-led training instead of taking full responsibility for increasing their trunk strength by themselves. Trunk exercise has been shown to decrease back pain [32–34]. Low back pain is one of the most common and debilitating musculoskeletal disorders [35, 36]. Chronic and intermittent disabling pain influences work ability, physical activity level, and need for health care and for medication [8, 37]. Possible prevention or reduction of back pain could lead to enormous positive effects for patients, health care and society [8, 38, 39].

All participants were clearly aware of how their physical function was being tested. The control group had to interpret their performance on their own and could very well have decided to try to improve on tests where they were dissatisfied with their results. While no numbers were provided to the control group, obvious difficulties may have inspired some to make lifestyle changes on their own which would make it difficult to interpret effects of the intervention. Clinically, it is no disadvantage if patients make appropriate lifestyle changes without counselling. However, results of larger studies will be necessary before it becomes apparent whether counselling sessions can be prioritised to sub-groups. All participants were also aware that their physical activity level was being measured. This may have influenced some to increase their activity level but, on the whole, the participants had a large amount of sedentary time and very little time in vigorous or very vigorous activity at both time points, so it seems unlikely that this had considerable effect.

Most people who set goals attained them wholly or partially. Most even followed their original plans set at the counselling sessions. According to participant feedback, knowing which dimensions needed improvement and knowing that they would be examined again contributed to success in making lifestyle changes [19]. That some people followed their plans for lifestyle changes without meeting their goals may reflect the short follow-up time or the fact that the participants had full responsibility for making lifestyle changes without any further support from health care or the research team.

As expected in this pilot study, there were no significant differences or clear tendencies between groups for change in health-related quality of life, self-assessed physical activity level or sedentary time, risk for developing chronic pain, or mental health. As the target group was relatively healthy middle-aged people, the participants had a fairly high health-related quality of life and low risk for developing chronic pain from the start. The project did not specifically aim to influence mental health, but levels of stress and anxiety did seem to change in a positive direction in the intervention group. Perhaps taking control of other health aspects can even help with certain kinds of mental health problems. It is difficult to interpret changes in self-assessed physical activity and sedentary time as there was so little correlation between self-assessed and objective values. The intervention group was given feedback on physical activity level based on self-assessed values from the questionnaires after the inclusion examinations. This may have led them to believe that their activity levels were better than they were. Overestimation of self-assessed physical activity level compared to objective values has been shown in previous research [40]. Both methods have strengths and weaknesses and could provide complemental information, even if accelerometer data is considered to be more reliable [17]. Adjusting the physical activity component of the risk profile to reflect the accelerometer values would be possible but would make implementation of the intervention into clinical practice more difficult.

Of those measures which are often followed regarding LRD – blood pressure, BMI, waist circumference, blood sugar, blood fats – none showed a clear effect of the intervention. This does not contradict the underlying hypothesis of this project that functional capacity can be seen as a forerunner to LRD and to commonly measured LRD risk factors. As functional capacity decreases, the risk for traditional LRDs which can be monitored by the above measures increases. It is possible for function to be influenced almost directly and with relatively limited interventions, while more traditional indicators may take longer time to both reach risk levels and to be influenced in positive directions by health care activities.

The effects of this intervention in both the short and long term need to be investigated on a larger scale. If good effects can be shown on better function, increased physical activity and other behavioural changes, and better understanding and control over factors which increase risk for LRD in the short term, perhaps long-term development of LRD can be avoided or postponed.

### Strengths and limitations

A strength of this study is that it tests a preventive risk-reducing intervention for LRD which participants found meaningful and motivating and which is possible to integrate into primary care routines [19]. It has a strong base in scientific literature and in earlier-tested preventive programs. In addition, it provides a foundation for planning future studies.

Limitations include the size of the study. It was not designed to find significant differences between groups but rather to gain a basic understanding of whether this new composite preventive intervention should be studied further and how future studies should be focused. The low recruitment rate resulted in a selected participant group who were physically inactive but highly motivated to improve their health situations and reduce risk for future LRD. This selection reduces the generalisability of the results. Recruitment barriers are discussed in our feasibility study and attempts will be made to overcome these in coming studies with increased cooperation with primary care centres and examination times outside of ordinary work hours [19].

## Conclusion

This small-scale pilot intervention with functional examinations and lifestyle counselling led to positive tendencies in short-term objectively measured physical activity level. It seemed to lead to better understanding of personal functional capacity and increased motivation for lifestyle changes. It contributed to participants setting and fulfilling goals for lifestyle-related changes which were meaningful to them and the functional levels on participants’ functional profiles were influenced in positive directions. The effects of this pilot study will be used in the design of a full-scale RCT.

## Supplementary Information

Supplementary Material 1.

Supplementary Material 2.

Supplementary Material 3.

## Data Availability

The datasets used and/or analysed during the current study are available from the corresponding author on reasonable request.

## Abbreviations

LRD: Lifestyle-Related Disorders
VIP: Västerbotten Intervention Programme
PAP: Physical Activity on Prescription
NBHW: (Swedish) National Board of Health and Welfare
NRS: Numerical Rating Scale
SCI-93: Stress and Crisis Inventory-93
HADS: Hospital Anxiety and Depression Scale
SD: Standard deviation
