# Supplementary material for: Function-based risk reduction intervention for lifestyle-related disorders among inactive 40-year-old people: a pilot randomised controlled trial
Source: BMC Public Health. 2024 Oct 13;24:2799. doi: 10.1186/s12889-024-20301-6 (PMC11479533; doi:10.1186/s12889-024-20301-6)
Supplement: Supplementary file 1 — Supplementary Material 1. [file 12889_2024_20301_MOESM1_ESM.docx]

Supplementary Table 1. Questionnaire results showing change between inclusion and follow-up.

| **Questionnaire** | **Intervention**  **n=13** | | | **Control**  **n=12** | | | **95% Confidence interval (between-groups change)** | **Interpretation.**  **Min-max. (reference)** |
| --- | --- | --- | --- | --- | --- | --- | --- | --- |
|  | *Inclusion*  *Mean (SD)* | *Follow-up*  *Mean (SD)* | *Mean change*  *(SD)* | *Inclusion*  *Mean (SD)* | *Follow-up*  *Mean (SD)* | *Mean change (SD)* |  |  |
| **Euroqol-5 dimensions-3L score** | 0.73  (0.32) | 0.73  (0.32) | 0.03  (0.08) | 0.82  (0.23) | 0.87  (0.20) | 0.05  (0.11) | -0.09, 0.07 | Health-related quality of life. Ca -0.50-1.00. (20) |
| **Euroqol-5 dimensions-3L VAS** | 66.93  (19.28) | 70.58  (18.09) | 5.75  (15.31) | 71.00  (19.92) | 73.50  (20.37) | 2.50  (4.38) | -6.70, 13.20 | Health-related quality of life. 0-100. (20) |
| **Saltin-Grimby Physical Activity Level Scale** | **2.20***  **(0.41)** | 2.25  (0.62) | 0.08  (0.67) | **1.75***  **(0.62)** | 1.92  (0.79) | 0.17  (0.72) | -0.67, 0.50 | Physical activity level. 0-4. (21) |
| **NBHW-Physical Activity** | **189.00***  **(128.64)** | 227.33  (121.19) | 22.33  (164.93) | **85.00***  **(62.12)** | 143.75  (119.22) | 58.75  (110.68) | -156.33, 83.49 | Physical activity level. 0-630. (22) |
| **SED-GIH** | 7.00  (2.93) | 6.08  (3.03) | -1.17  (2.33) | 8.17  (4.15) | 8.67  (3.89) | 0.50  (2.50) | -3.71, 0.38 | Self-reported daily sedentary time (hrs). 0-24. (23) |
| **ÖMPSQ-short form** | 34.20  (19.51) | 35.50  (13.06) | 1.00  (12.28) | 25.25  (19.44) | 26.42  (17.80) | 1.17  (9.29) | -9.43, 9.09 | Risk for developing chronic pain. 0-100. (24) |
| **Motivation** | 7.67  (1.80) | 8.25  (1.14) | 1.00  (1.54) | 7.00  (2.66) | 6.42  (2.94) | -0.58  (1.73) | **0.20, 2.97** | Motivation for lifestyle change. 0-10. (19) |
| **Stress and Crisis Inventory-93** | 25.67  (19.72) | 22.42  (18.98) | -4.33  (11.36) | 17.67  (22.67) | 19.83  (26.44) | 2.17  (9.03) | -15.22, 2.22 | Stress symptoms. 0-140. (25) |
| **Hospital Anxiety and Depression Scale-Anxiety** | 7.20  (4.31) | 5.92  (3.94) | -2.08  (4.23) | 4.75  (4.77) | 3.92  (4.19) | -0.83  (2.72) | -4.29, 1.79 | Anxiety symptoms. 0-21. (26) |
| **Hospital Anxiety and Depression Scale-Depression** | 4.13  (2.88) | 3.42  (3.09) | -1.17  (3.41) | 4.17  (4.11) | 3.75  (5.08) | -0.42  (2.68) | -3.35, 1.85 | Depression symptoms. 0-21. (26) |
| **Self-reported any illness** | 0.27  (0.46) |  |  | 0.33  (0.49) |  |  | -0.45, 0.32^a^ | Proportion participants reporting any illness. |
| **Self-reported depression** | 0.00  (0.00) |  |  | 0.17  (0.39) |  |  | -0.41, 0.08^a^ | Proportion participants reporting depression. |

*Significant difference between groups at inclusion, 95%CI (0.01, 0.89) for SGPALS and (25.52, 182,48) for NBHW-PA. ^a^p at inclusion. VAS=Visual Analogue Scale; NBHW=(Swedish) National Board of Health and Welfare; ÖMPSQ=Örebro Musculoskeletal Pain Screening Questionnaire.
