# Supplementary material for: Function-based risk reduction intervention for lifestyle-related disorders among inactive 40-year-old people: a pilot randomised controlled trial
Source: BMC Public Health. 2024 Oct 13;24:2799. doi: 10.1186/s12889-024-20301-6 (PMC11479533; doi:10.1186/s12889-024-20301-6)
Supplement: Supplementary file 2 — Supplementary Material 2. [file 12889_2024_20301_MOESM2_ESM.docx]

Supplementary Table 2. Functional test results showing change between inclusion and follow-up.

| **Functional test** | **Intervention**  **n=13** | | | **Control**  **n=12** | | | **95% Confidence interval (between-groups change)** |
| --- | --- | --- | --- | --- | --- | --- | --- |
|  | *Inclusion*  *Mean*  *(SD)* | *Follow-up*  *Mean*  *(SD)* | *Mean change*  *(SD)* | *Inclusion*  *Mean*  *(SD)* | *Follow-up*  *Mean*  *(SD)* | *Mean change*  *(SD)* |  |
| **Ekblom-Bak ergometer (mL/kg/min)** | 34.1  (6.5) | 35.5  (7.7) | 0.7  (3.3) | 33.8  (8.8) | 34.3  (6.4) | 0.5  (4.2) | -3.05, 3.46 |
| **2-minute Step-test (n)** | 123.3  (25.2) | 142.5  (33.5) | 21.5  (25.4) | 131.1  (29.3) | 141.3  (28.4) | 10.2  (13.1) | -6.12, 28.79 |
| **Handgrip Strength^a^ (kg)** | 46.2  (13.6) | 45.2  (14.6) | -0.8  (2.8) | 41.6  (15.5) | 42.1  (16.9) | 0.5  (3.4) | -3.95, 1.37 |
| **Biceps (n)** | 22.1  (5.3) | 24.0  (6.4) | 1.5  (4.5) | 21.0  (7.7) | 23.8  (6.9) | 2.8  (3.4) | -4.61, 2.11 |
| **30s Chair-stand (n)** | 17.7  (5.2) | 21.0  (5.2) | 3.2  (2.7) | 16.1  (5.4) | 19.0  (6.4) | 2.9  (3.5) | -2.39, 2.89 |
| **Single-foot Heel Rises^a^ (n)** | 20.4  (6.2) | 20.9  (7.8) | 0.1  (4.9) | 17.0  (5.8) | 19.2  (6.6) | 2.2  (5.3) | -6.45, 2.20 |
| **Plank (s)** | 78.7  (43.7) | 81.4  (45.5) | 4.2  (12.7) | 80.2  (67.9) | 65.9  (51.3) | -14.3  (19.7) | **4.25, 32.58** |
| **Back Endurance (s)** | 73.1  (58.1) | 67.0  (33.0) | -1.2  (30.6) | 67.9  (47.9) | 66.4  (38.6) | -1.5  (21.2) | -22.13, 22.79 |
| **Supine Bridge (s)** | 142.8  (42.7) | 158.1  (66.0) | 17.4  (41.3) | 141.3  (65.0) | 138.0  (49.8) | -3.3  (23.1) | -8.14, 49.47 |
| **SOLEO^b^ (s)** | 132.6  (59.3) | 151.4  (56.4) | 30.7  (43.2) | 115.3  (65.1) | 125.0  (66.6) | 9.7  (27.7) | -10.03, 52.03 |
| **SOLEC (s)** | 27.5  (46.9) | 15.7  (9.8) | -3.5  (28.8) | 11.7  (13.7) | 8.3  (5.0) | -3.4  (10.2) | -19.03, 18.86 |
| **Functional Reach (cm)** | 39.6  (10.7) | 40.2  (10.3) | 1.5  (10.7) | 36.2  (12.1) | 39.7  (9.7) | 3.5  (6.4) | -96.39, 55.55 |
| **Lateral Reach^a^ (cm)** | 23.2  (4.6) | 24.6  (5.1) | 1.6  (2.7) | 24.0  (6.9) | 22.6  (7.2) | -1.4  (5.1) | -0.55, 6.47 |
| **Sharpened Romberg^b^ (s)** | 40.8  (21.3) | 40.8  (22.2) | 1.8  (18.2) | 33.2  (23.2) | 36.7  (23.0) | 3.5  (23.3) | -19.41, 16.07 |
| **Sit-Rise (poäng)** | 8.9  (1.0) | 9.3  (0.9) | 0.7  (0.8) | 9.1  (1.1) | 9.3  (0.7) | 0.1  (0.7) | -0.09, 1.17 |
| **Finger-Floor (cm)** | 5.3  (7.1) | 4.5  (6.9) | -1.8  (5.6) | 9.2  (7.4) | 8.0  (7.3) | -1.2  (3.6) | -4.63, 3.47 |
| **Lateral Flexion^a^ (cm)** | 15.4  (8.9) | 22.6  (64.1) | 6.5  (12.3) | 17.9  (7.2) | 19.4  (6.4) | 1.0  (9.7) | -40.93, 150.48 |
| **BHS (poäng)** | 1.9  (2.2) | 2.3  (2.9) | 0.8  (1.8) | 2.5  (1.8) | 2.0  (1.8) | -0.5  (1.5) | -0.17, 2.67 |
| **Occiput-to-Wall (poäng)** | 0.9  (0.4) | 0.9  (0.3) | 0.1  (0.5) | 0.9  (0.3) | 0.8  (0.4) | -0.1  (0.5) | -0.27, 0.60 |
| **Navicular Drop^a^ (mm)** | 4.1  (1.5) | 2.4  (1.5) | -1.5  (1.9) | 5.4  (3.7) | 3.0  (2.3) | -2.4  (4.3) | -2.03, 3.69 |
| **Patella Mobility^a^ (poäng)** | 0.4  (0.5) | 0.4  (0.4) | 0.0  (0.3) | 0.3  (0.4) | 0.3  (0.4) | 0.0  (0.3) | -0.31, 0.23 |

^a^Mean value for left and right is used for bilateral tests. ^b^Maximum value creates ceiling effect in some cases. SD=Standard deviation; SOLEO=Stand-on-one-leg-eyes-open; SOLEC=Stand-on-one-leg-eyes-closed; BHS=Beighton Hypermobility Score. Details of all tests previously published (19).
