# Supplementary material for: Function-based risk reduction intervention for lifestyle-related disorders among inactive 40-year-old people: a pilot randomised controlled trial
Source: BMC Public Health. 2024 Oct 13;24:2799. doi: 10.1186/s12889-024-20301-6 (PMC11479533; doi:10.1186/s12889-024-20301-6)
Supplement: Supplementary file 3 — Supplementary Material 3. [file 12889_2024_20301_MOESM3_ESM.docx]

Supplementary Table 3. Results of medical examinations showing change between inclusion and follow-up.

| **Measure** | **Intervention**  **n=13** | | | **Control**  **n=12** | | | **95% Confidence interval (between-groups change)** |
| --- | --- | --- | --- | --- | --- | --- | --- |
|  | *Inclusion Mean*  *(SD)* | *Follow-up*  *Mean*  *(SD)* | *Mean change*  *(SD)* | *Inclusion Mean*  *(SD)* | *Follow-up*  *Mean*  *(SD)* | *Mean change*  *(SD)* |  |
| **Weight (kg)** | 80.4  (14.1) | 80.7  (15.1) | 0.7  (2.7) | 73.7  (13.3) | 74.1  (13.2) | 0.4  (2.2) | -1.77, 2.45 |
| **Body mass index (kg/m^2^)** | 27.1  (4.2) | 27.5  (5.2) | 0.2  (1.0) | 24.1  (2.7) | 24.3  (2.7) | 0.1  (0.7) | -0.60, 0.84 |
| **Waist circumference (cm)** | 92.4  (11.0) | 92.6  (13.3) | -0.4  (2.9) | 88.1  (10.4) | 89.2  (10.9) | 1.0  (4.0) | -4.40, 1.48 |
| **Systolic blood pressure (mmHg)** | 121.9  (10.0) | 125.6  (13.9) | 5.0  (14.2) | 115.7  (15.4) | 115.2  (20.4) | -0.5  (9.4) | -4.77, 15.77 |
| **Diastolic blood pressure (mmHg)** | 80.7  (6.7) | 81.2  (8.9) | -0.2  (8.5) | 81.0  (11.8) | 79.3  (12.7) | -1.7  (7.0) | -5.09, 8.09 |
| **Glucose (mmol/L)** | 5.0  (0.7) | 5.2  (0.4) | 0.3  (0.6) | 5.3  (0.6) | 5.3  (0.3) | 0.0  (0.5) | -0.15, 0.74 |
| **Total cholesterol (mmol/L)** | 4.8  (0.9) | 5.0  (1.0) | 0.3  (0.6) | 4.2  (0.9) | 4.6  (1.3) | 0.4  (0.7) | -0.64, 0.40 |
| **Triglycerides (mmol/L)** | 1.5  (1.0) | 1.6  (1.2) | 0.1  (1.2) | 0.9  (0.4) | 0.8  (0.3) | -0.1  (0.4) | -0.52, 0.94 |
| **LDL (mmol/L)** | 3.2  (0.9) | 3.4  (1.0) | 0.3  (0.7) | 2.7  (1.0) | 3.2  (1.3) | 0.4  (0.6) | -0.70, 0.38 |
| **HDL (mmol/L)** | 1.2  (0.3) | 1.3  (0.3) | 0.1  (0.1) | 1.3  (0.3) | 1.4  (0.3) | 0.1  (0.2) | -0.15, 0.13 |
| **LDL/HDL** | 2.7  (1.0) | 2.8  (1.1) | 0.1  (0.5) | 2.3  (1.1) | 2.4  (1.0) | 0.1  (0.4) | -0.36, 0.32 |
| **Smoker (n)** | 2 | 1 | 1 | 1 | 1 | 0 |  |

LDL=low density lipoproteins; HDL=high density lipoproteins; SD=Standard deviation.
